# The effect of montages of transcranial alternating current stimulation on occipital responses—a sham-controlled pilot study

**DOI:** 10.3389/fpsyt.2023.1273044

**Published:** 2024-01-24

**Authors:** Jingying Wang, Kai Yip Choi, Benjamin Thompson, Henry Ho Lung Chan, Allen Ming Yan Cheong

**Affiliations:** ^1^School of Optometry, The Hong Kong Polytechnic University, Hong Kong, Hong Kong SAR, China; ^2^College of Health and Human Performance, University of Florida, Gainesville, FL, United States; ^3^School of Optometry and Vision Science, University of Waterloo, Waterloo, ON, Canada; ^4^Centre for Eye and Vision Research, Hong Kong Science Park, Hong Kong, Hong Kong SAR, China

**Keywords:** non-invasive brain stimulation, transcranial alternating current stimulation, transcranial electrical stimulation, montage, occipital excitability, visual evoked potentials

## Abstract

**Background:**

Transcranial alternative current stimulation (tACS) refers to a promising non-invasive technique to improve brain functions. However, owing to various stimulation parameters in the literature, optimization of the stimulation is warranted. In this study, the authors aimed to compare the effect of tACS electrode montages on occipital responses.

**Methods:**

In three montage sessions (i.e., Oz-Cz, Oz-cheek, and sham), 10 healthy young adults participated, receiving 20-min 2-mA alpha-tACS. Pattern-reversal visual evoked potentials (VEPs) were measured before tACS (T0), immediately after (T20), and 20 min (T40) after tACS. Normalized changes in time-domain features (i.e., N75, P100 amplitudes, and P100 latency) and frequency-domain features [i.e., power spectral density in alpha (PSDα) and beta (PSDβ) bands] were evaluated.

**Results:**

In contrast to our hypothesis, the occipital response decreased immediately (T20) after receiving the 20-min tACS in all montages in terms of P100 amplitude (*p* = 0.01). This reduction returned to baseline level (T0) in Oz-cheek and sham conditions but sustained in the Oz-Cz condition (T40, *p* = 0.03) after 20 min of tACS. The effects on N75 amplitude and P100 latency were statistically insignificant. For spectral analysis, both PSDα and PSDβ were significantly increased after tACS at T20, in which the effect sustained until T40. However, there was no differential effect by montages. There was no significant difference in the occurrence of sensations across the montages. The effectiveness of the blinding is supported by the participants’ rate of guessing correctly.

**Conclusion:**

This study revealed an immediate inhibitory effect of tACS, regardless of the montages. This inhibitory effect sustained in the Oz-Cz montage but faded out in other montages after 20 min.

## 1 Introduction

Transcranial electrical stimulation (tES), encompassing a range of techniques that deliver low-intensity electricity to the brain, has been substantially studied for its effect on brain functions in healthy people and for its therapeutic effects on clinical symptoms in those with psychiatric disorders (1, 2). The therapeutic and remedial effects of tES arise from both acute and long-term neuroplastic alterations in cortical excitability at the macroscopic level, triggered by alterations in brain activity (3). Increasing evidence has demonstrated that the tES targeting on the occipital region (Oz) can enhance, preserve, and even restore visual functions in healthy and visually impaired individuals (4). However, the effects of tES on vision remain inconclusive in the literature [refer to (5) for a recent systematic review]. This variability may stem from diverse stimulation parameters employed across studies, influencing the neuroplastic aftereffects and the subsequent efficacy of tES. Such parameters include, but not limited to, current density, polarity, stimulation duration, and/or geometrical montage of electrodes (6–9). Adjusting these parameters can lead to divergent outcomes in tES effects, including excitation versus inhibition and variations in the persistence of the aftereffect.

The location of the tES electrodes is crucial to determine the cortical area to be modulated and the amount of electrical current to be delivered (6, 10)—active electrode: maximal intracranial effect (11); reference electrode: tissue polarization (12). Regarding the visual system, the effects of transcranial direct current stimulation (tDCS) vary depending on the position of the reference electrode (13). Kraft et al. argued that the post-tES change in the amplitudes of visual evoked potentials (VEPs) reported in Antal et al. were in the opposite direction to that reported in Accornero et al. due to the use of different montages (Oz-Cz vs. Oz-posterior neck base) (7, 10, 13). Reinhart et al. applied Oz-cheek montage and found that tDCS had a larger and longer effect on enhancing visual acuity than that reported in Antal et al. using Oz-Cz montage (14, 15).

In addition to tDCS, transcranial alternating electrical stimulation (tACS) is another mode of tES, which employs specific-frequency electrical stimulation to the brain (16). With tDCS, neuronal firing rates vary with the polarity of direct current due to induced changes in resting membrane potentials (17). Unlike tDCS, tACS has no fixed anode/cathode as the polarity alternates during a half cycle of tACS oscillation; instead, it modulates the wave rate to entrain specific brain rhythm (18). Effects of tACS on visual functions were mixed. On one hand, tACS was found to impair visual detection (19, 20) and temporal resolution (21). On the other hand, it was found to be effective in improving the contrast sensitivity function (22). In terms of visual electrophysiology, tACS was found to increase the amplitude of the steady-state VEP when the frequencies of the tACS and VEP were matched (23).

tACS has been suggested to be superior in modulating brain functions than other modes of tES, given that the stimulating brain areas are the same. According to Manoli et al., 10-Hz tACS was suggested to generate a stronger and more focused effect than tDCS (24). Inukai et al. also found a significant increase in motor evoked potentials with tACS, but not with tDCS, when compared with sham measurements (25). Recent studies demonstrated that tACS directly boosted visual perceptual learning (26). In contrast, tDCS only enhanced visual perceptual learning performance when the subjects had good sleep quality (27).

Several studies also have investigated the effects of tACS by varying its parameters. A recent study by Wang et al. discovered that both the current intensities and the condition of eyes being open or closed have an impact on occipital excitability, with 2 mA in the eyes-open condition inducing notably higher alpha activity (28). Additionally, the selection of the electrode montage, specifically the placement of electrodes, can significantly alter the outcomes of tACS. Hsu et al. reported that facial stimulation montage predominantly produced phosphenes in the upper-left visual field, whereas using the occipital montage led to a more uniform distribution of phosphenes throughout the visual field (29). These studies inspired us to further investigate optimizing these parameters to achieve desired neural outcomes.

Summarizing the above evidence, the stimulation parameters might be a factor in the variation of tES outcomes among studies; hence, optimization is warranted. In this study, we aimed to compare the effects of two tACS montages on occipital excitability. We hypothesized that the Oz-Cz gave a better excitability than the Oz-cheek montage, in terms of amplitudes of VEP, and a better entrainment effect in terms of a longer maintained log difference in power spectral density analyses.

## 2 Materials and methods

This study adopted a single-blind sham-controlled crossover design and was conducted from September to November 2021. All procedures followed the tenets of the Declaration of Helsinki and were approved by the Institutional Review Board of The Hong Kong Polytechnic University (HSEARS20210713002).

### 2.1 Participants

The inclusion criteria included the following: (1) adults of age > 18 years; (2) normal or corrected-to-normal vision (LogMAR 0.00 or equivalent); (3) no contraindications to receive tES (e.g., no wounds on the scalp); (4) no history of any neuropsychiatric disorder; and (5) no prior history of drug abuse or alcoholism. Participants were not allowed to take any medication or stimulating foods/drinks (e.g., alcohol, coffee, tobacco, and chocolate) at least 24 h before the sessions. All participants signed an informed consent form and a modified safety checklist from the Transcranial Magnetic Stimulation Adult Safety Screen (TASS) (Supplementary Appendix 1) (30) before taking part in the study.

### 2.2 Pattern-reversal visual evoked potential

Pattern-reversal visual evoked potential (VEP) was applied to measure the occipital responses (Diagnosys Espion, LLC, United States), following a previous version of the ISCEV standard (31). VEP stimuli were displayed on a cathode-ray tube monitor (CM715, Hitachi, Japan) as a 99% contrast black (1 cd/m^2^) and white (100 cd/m^2^) checkerboard with 1.5 Hz temporal frequency, i.e., 3.0 reversals per second (rps) at a viewing distance of 1 m. The current standard for pattern reversal is 2.0 rps (32), but a slightly higher 3.0 rps was chosen because of the proximity to the alpha band without triggering a steady-state response (31). The recording position of VEP was located at Fpz (reference) and Oz (active) with grounding on the mastoid bone of the ipsilateral side. The position of Oz was confirmed by measuring the distance D (cm) between the nasion and inion and was located at 10% D above the inion. In each measurement, VEP was tested at least twice to ensure good repeatability for each pattern spatial frequency, i.e., check size with a subtended visual angle of 1.0° and 0.25°, and 75 trials were obtained in each block with the blinked traces removed by a masked observer. The impedance was controlled under 5 kΩ during the whole experiment.

### 2.3 Transcranial alternating current stimulation

Transcranial alternating current stimulation (tACS) was delivered using a Nurostym (Brainbox, United Kingdom) to modulate the occipital responses. The size of the electrodes was 5 × 5 cm, and the tACS parameters were 2 mA, 10 Hz, for 20 min per session, with a 30-s fade in/out. Based on the 10–20 system, two montages—Oz-Cz (occipital and central midline) and Oz-cheek—were selected and their effects on VEP were compared. For the Oz-cheek montage, the cheek side was contralateral to the randomly selected test eye of VEP. For the sham stimulation, the montage was selected as Oz-Cz or Oz-cheek randomly at a ratio of 1:1. Sham consisted of an active stimulation delivered for 30 s to mimic the initial sensation of active tACS, acting like a placebo to mask the participants. Impedance was controlled below 10 kΩ for tACS electrodes.

### 2.4 Procedures

**Figure 1:**
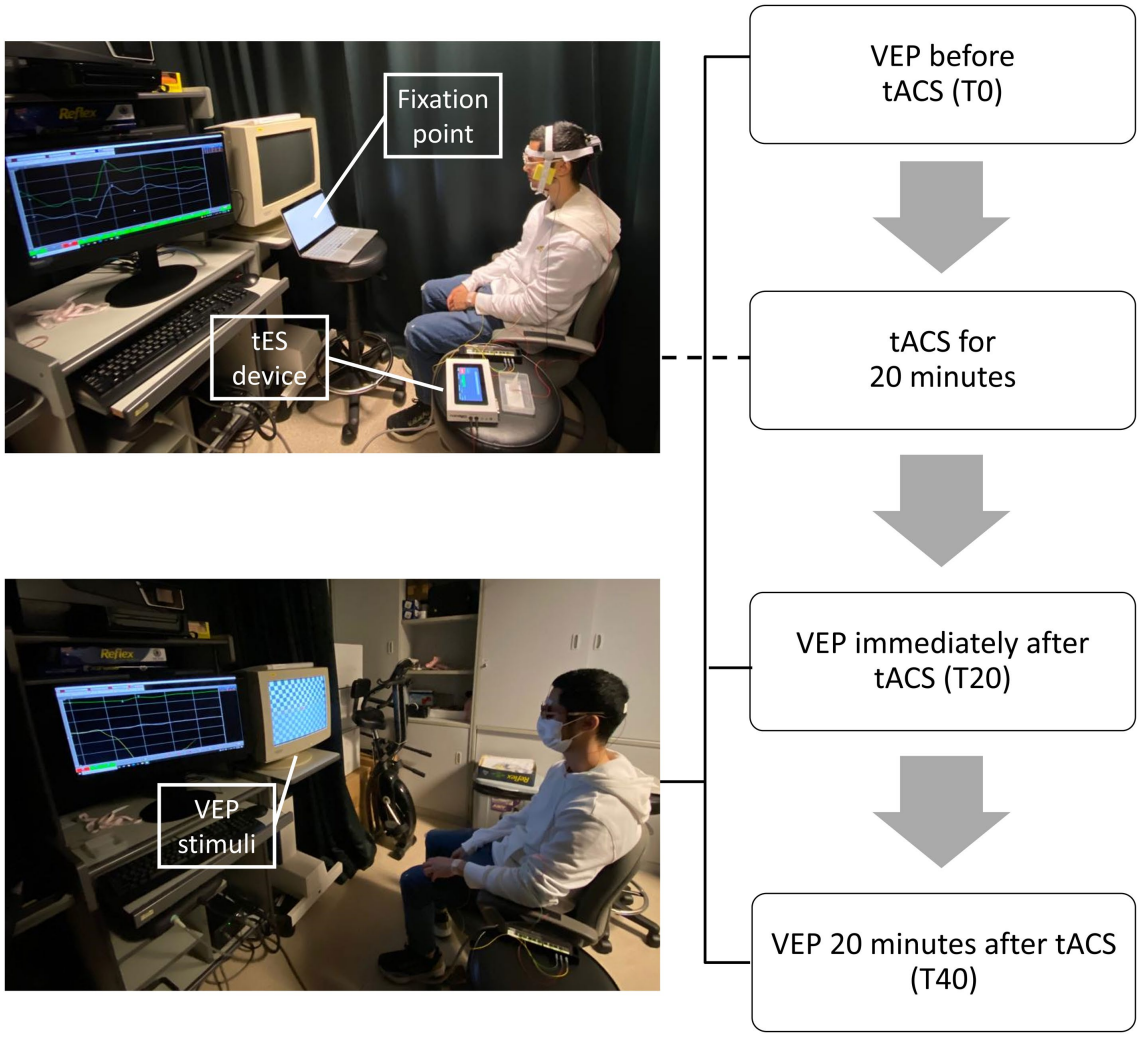
Procedures of each session.

All participants took part in all three conditions (Oz-Cz, Oz-cheek, and sham) in a random sequence. For the Oz-Cz and Oz-cheek conditions, the tACS electrode was placed on Oz, while the reference electrode was placed on Cz and cheek, respectively. Sham was one of the two montage positions for each participant using a randomized way. For each condition, VEPs were measured pre-stimulation (T0), immediately after (T20), and 20 min after (T40) tACS in one randomly selected test eye (Figure 1). The duration of each session was approximately 1.5 h, including setting up the equipment and positioning the stimulation electrodes. At the end of the session, participants were asked to subjectively grade their sensations on a scale of 0–4 and speculate whether they had received an active or sham stimulation. The washout period between the two trials was a minimum of 24 h.

Online measurement (i.e., VEPs during tACS) was not adopted because of the difficulty of analyzing the acquired neural responses at the same frequency as the stimulation applied, that is, cortical enhancement in alpha oscillations during the 10-Hz tACS stimulation (33).

### 2.5 Data processing

To reduce the noises, including eye movements (e.g., blinks and ocular versions), myoelectricity (derived from eyes, head, and forehead), and power frequencies (e.g., powerline hum and radio frequency), a bandpass filter of 0.03–30 Hz was set up as the configuration. VEPs were automatically cut into epochs in the software, and epochs beyond ±800 mV were rejected by a masked observer, in which 60–75 epochs were then averaged to obtain the mean VEP. Time- and frequency-domain features were extracted from the averaged VEP epochs. From the two repeatable waveforms in each check size and stimulation condition, the first trough at approximately 75 ms was marked as N75 (amplitude defined as the difference of trough from zero), and the first peak at approximately 100 ms was marked as P100 (amplitude defined as the difference of peak from N75 trough). The latency was defined as the time at P100. For the frequency-domain features, both the power spectral density values (in mV2/Hz) in alpha (8–12 Hz, PSDα) and beta (12–30 Hz, PSDβ) bands were computed by averaged VEPs using fast Fourier transform with the Welch method (34). The normalized changes of VEP from T0 to T20 and T40 were calculated in percentage, which represented the effect of tACS on the averaged VEP responses:


$$\mathrm{Normalized}\;\%\;\mathrm{Change}=100\times \frac{{\bar{VEP}}_{T20\;\mathrm{or}\;T40}-{\bar{VEP}}_{T0}}{{\bar{VEP}}_{T0}}$$

### 2.6 Statistical analysis

All statistical procedures were performed using SPSS 26 (IBM, United States). To evaluate the main effects of the stimulus size, time, and montages, three-way repeated-measures ANOVAs on the absolute values of the VEP features were used, while two-way repeated-measures ANOVAs (3 montages * 2 sizes) were used to compare among different montages using the normalized changes. Except P100 amplitude, time-domain VEP features (N75 amplitude and P100 latency) violated the assumption of normal distribution in parametric analyses. Hence, the results were transformed to achieve the normality using percentile ranking followed by an inverse-normal transformation into Z-scores (35). For spectral domain analysis, the PSDα and PSDβ were logarithmically transformed to achieve normal distribution, and the logarithmic difference was analyzed. Holm’s adjustment was applied in post-hoc comparisons (36). Partial eta-squared (η^2^_p_) was reported as the effect size, which is a metric reflecting the percentage of the variance in the dependent variable explained by the independent variables. For η^2^_p_: small effect = 0.01; medium effect = 0.06; and large effect = 0.14. Bayes factors were also reported to represent the odds of the alternative hypotheses over the null hypotheses (37). Chi-squared tests were used to evaluate the occurrence of subjective sensations and the accuracy of speculating an active stimulation.

## 3 Results

All the experimental procedures were completed by 10 normally sighted (corrected LogMAR ≤0.00) adults (6 women, aged 28.2 ± 7.7 years). The impedance caused by tACS in Oz-cheek montage was smaller (8.85 ± 1.22 Ω) than that of Oz-Cz montage (10.6 ± 1.63 Ω). Percentage change in P100 amplitude in all measurements followed a normal distribution (Shapiro–Wilk test, all *p* > 0.18). After normality transformation, the Shapiro-Wilk test showed an approximately normal distribution for normalized changes in N75 amplitude (all *p* > 0.13), P100 latency (all *p* > 0.07), log PSDα (all *p* > 0.14), and log PSDβ (all *p* > 0.14).

### 3.1 Time-domain features

**Figure 2:**
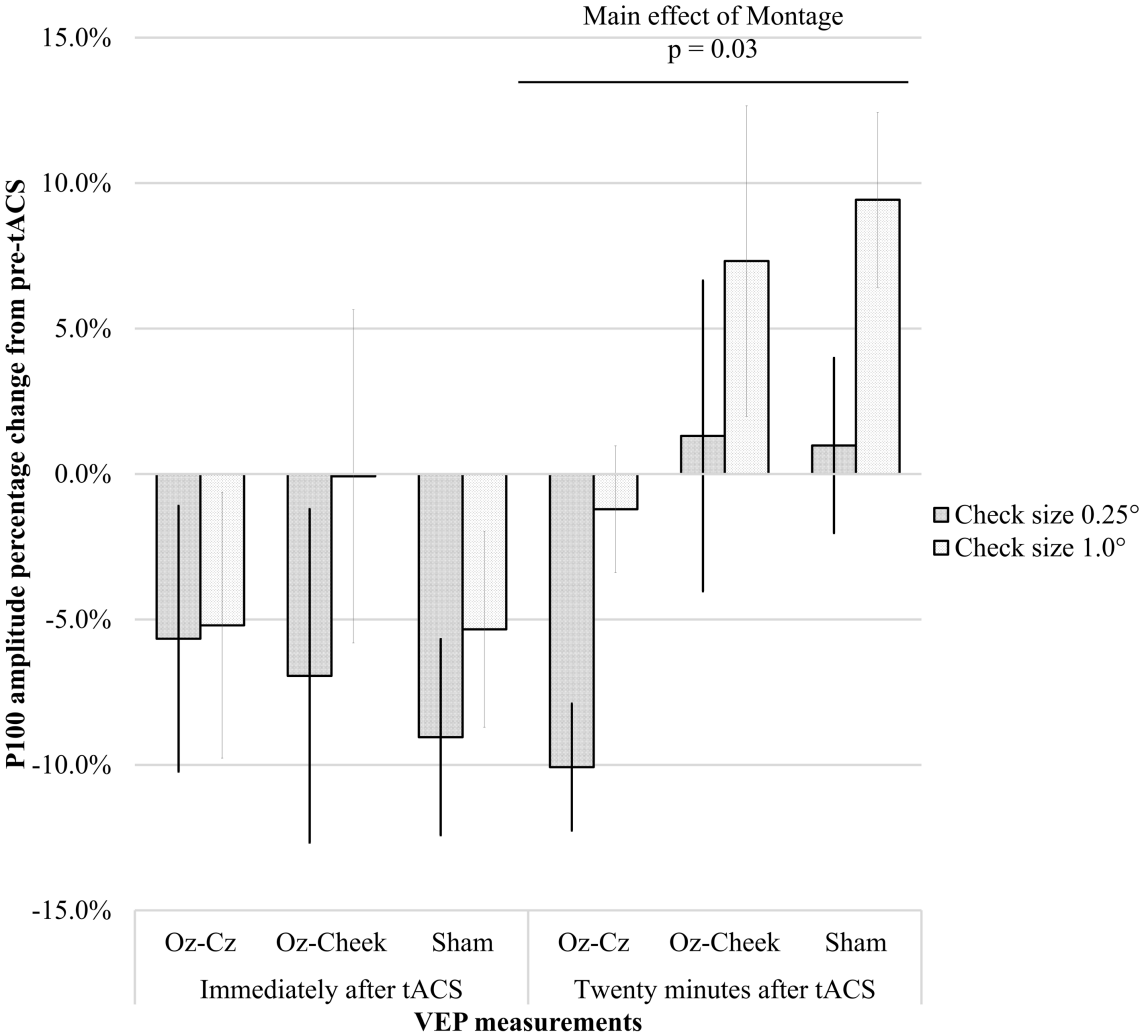
Percentage changes in P100 amplitude immediately after tACS (T20) and 20 min after tACS (T40) for different montages. Error bars represent the standard errors of mean.

**Figure 3:**
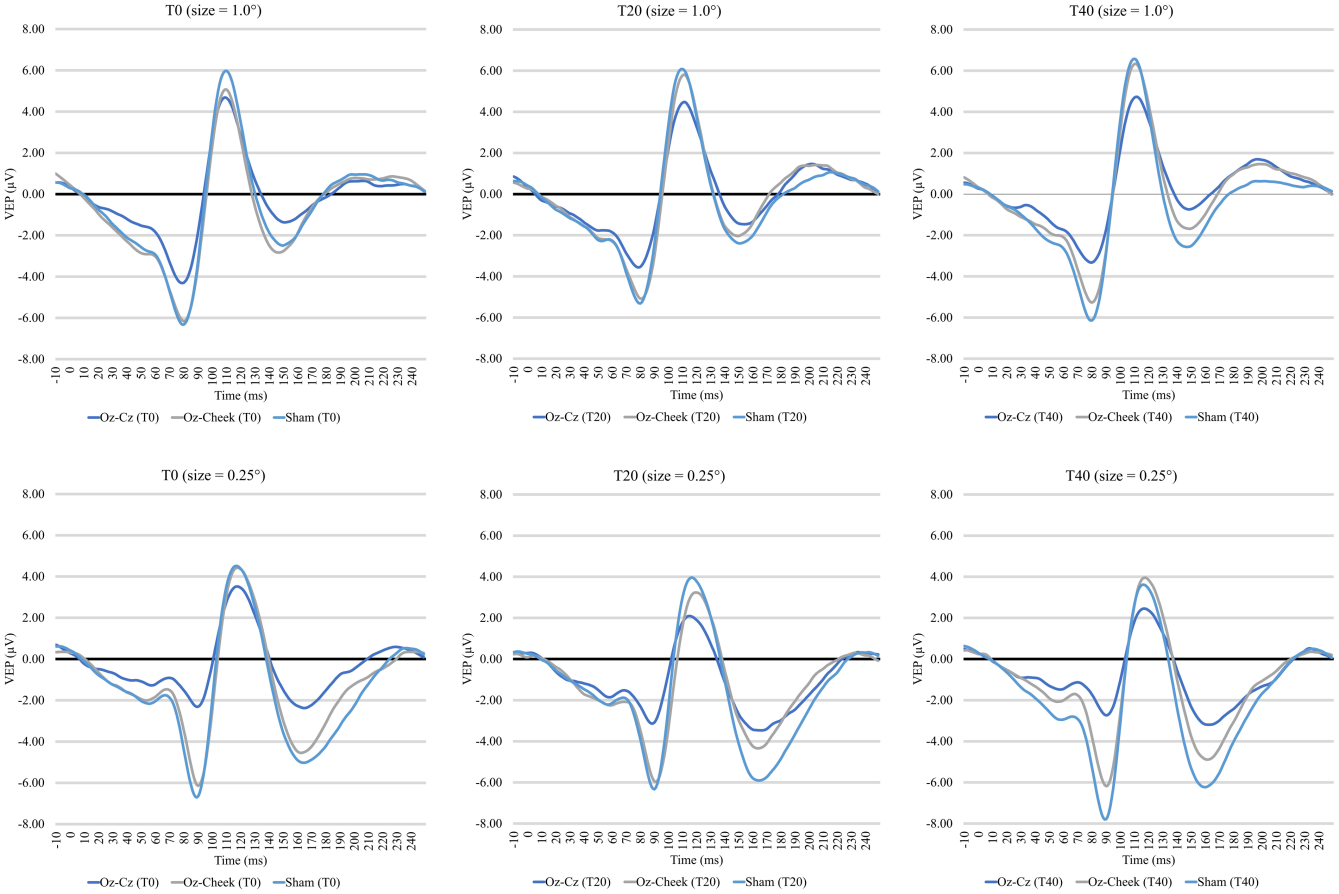
Time-domain visual evoked potentials at three time points for two check sizes. Top panel: 1.0 degree, bottom panel: 0.25 degree.

Supplementary Table S1 shows the time-domain features, including N75 amplitude, P100 amplitude, and latency of the VEP measurements before and after the tACS, while the results of repeated-measures ANOVA on normalized changes of VEP features are summarized in Table 1. For N75 amplitude and P100 latency, the main effects of the montage and size, as well as their interaction, were all insignificant on normally transformed percentage changes immediately and 20 min after the tACS (*p* > 0.09). For P100 amplitude, the effects were also insignificant immediately after tACS. However, the main effects of both the montage and size were significant at T40 (*p* = 0.01 and *p* = 0.03, respectively). In the post-hoc comparison, Oz-Cz montage was significantly smaller than that of sham stimulation after Holm’s adjustment (*p* < 0.05), but other pairs of montage were similar. Figure 2 shows the percentage change in P100 amplitude immediately and 20 min after tACS, stratified by montages and stimulus sizes, while Figure 3 demonstrates the waveforms obtained from VEP in different conditions.

**Table 1:** Results of two-way repeated-measures ANOVA in normalized percentage changes of VEP time-domain features and logarithmic difference of spectral-domain features.

	N75 amplitude^*^			P100 amplitude			P100 latency^*^			Log α-band PSD			Log β-band PSD		
	*p*	*η* ^2^ *_p_*	BF_10_	*p*	*η* ^2^ *_p_*	BF_10_	*p*	*η* ^2^ *_p_*	BF_10_	*p*	*η* ^2^ *_p_*	BF_10_	*p*	*η* ^2^ *_p_*	BF_10_
	T20—immediately after tACS														
Montage (M)	0.67	0.04	0.18	0.68	0.04	0.17	0.60	0.06	0.31	0.15	0.19	0.53	0.15	0.19	0.58
Size (S)	0.40	0.08	0.39	0.16	0.20	0.41	0.65	0.02	0.33	0.06	0.35	1.41	0.09	0.28	1.22
M * S	0.81	0.02	n/a	0.86	0.02	n/a	0.48	0.08	n/a	0.63	0.05	n/a	0.63	0.05	n/a
M + S	n/a	n/a	0.07	n/a	n/a	0.07	n/a	n/a	0.11	n/a	n/a	0.77	n/a	n/a	0.69
M + S + M * S	n/a	n/a	0.02	n/a	n/a	0.02	n/a	n/a	0.03	n/a	n/a	0.25	n/a	n/a	0.22
	T40—20 min after tACS														
Montage (M)	0.33	0.12	0.36	**0.03**	**0.32**	4.67	0.52	0.07	0.32	0.11	0.22	0.97	0.12	0.21	0.97
Size (S)	0.09	0.29	0.42	**0.01**	**0.61**	3.02	0.23	0.16	3.47	0.14	0.23	0.81	0.16	0.21	0.77
M * S	0.82	0.02	n/a	0.92	0.01	n/a	0.25	0.14	n/a	0.83	0.02	n/a	0.77	0.03	n/a
M + S	n/a	n/a	0.16	n/a	n/a	21.05	n/a	n/a	1.20	n/a	n/a	0.81	n/a	n/a	0.75
M + S + M * S	n/a	n/a	0.04	n/a	n/a	4.88	n/a	n/a	0.70	n/a	n/a	0.39	n/a	n/a	0.20

Bold indicates statistically significant results. ^*^Transformed by inverse-normal transformation. PSD, power spectral density. For *η*^2^_*p*_: small effect = 0.01; medium effect = 0.06; and large effect = 0.14. BF_10_: <1, results favor null hypothesis; 1–3, anecdotal; 3–10, moderate evidence; 10–30, strong evidence; 30–100, very strong evidence; >100, extreme evidence.

### 3.2 Frequency-domain features

The logarithmic PSD values, as shown in Supplementary Table S1, of both α- and β-bands all increased after tACS for all montages at T20 and retained until T40. However, neither the main effect of the montage or size nor the interaction was significant in either T20 or T40 (*p* > 0.06) for both α- and β-bands; the statistical results are summarized in Table 1.

### 3.3 Subjective sensation grading

In all tACS montages, 90% of the participants reported at least one type of mild sensation in any of the montages. Stinging, phosphene, and itchiness were the most frequently reported sensations, as shown in Supplementary Figure S1. Results from the chi-square test did not reveal a significant difference in the occurrence of subjective sensation between montages (*p* > 0.12). As for the grading, almost all reported well tolerated (Score = 1 or 2), except for one grade 3 and another grade 4 itchiness reported during Oz-Cheek stimulation. The chi-square test also demonstrated similar accuracies of speculation for different montages (*p* = 0.35), supporting the blinding for participants was valid.

## 4 Discussion

The current study compared the effect of tACS at the occipital cortex using different montages on VEP. Our results revealed an immediate inhibitory effect of tACS on P100 amplitude, in which the effect sustained for 20 min after stimulation in the Oz-Cz montage. However, tACS did not have a significant effect on N75 amplitude or P100 latency. In addition, the entrainment effect was inconclusive from the PSD analysis.

### 4.1 Time-domain results

Our results demonstrated that P100 amplitude was significantly decreased by either stimulating Oz-Cz or Oz-cheek. However, the difference between active and sham stimulations did not happen as expected. From Figure 2, considering the similar trends of P100 amplitude over time in all the montages, the small sample size in the current study could be a possible reason, which was inadequate to identify the essential difference between the active and sham groups. Another possibility was that the tACS intensity adopted in the current study was too weak to alter the cortical response in humans. Vöröslakos et al. suggested applying at least 4–6 mA currents through the conventional tACS electrodes for modulating the amplitude of alpha waves reliably and instantaneously in human subjects due to the fact that only approximately 25% of the scalp-applied current would be able to enter the brain transcranially (11). However, the efficacy and tolerability of current intensity larger than 4 mA are still in doubt, especially for tACS. De Koninck et al. reported that 1-mA tACS induced stronger aftereffects in alpha power than that stimulated by 4–6 mA (38).

Another interesting finding was that the 10-Hz tACS played an inhibiting role in occipital excitability, which was opposing to our expectation. This inhibitory role was consistently demonstrated in a recent study (39), in which the tDCS significantly reduced the P100 amplitude of the occipital cortex. Other earlier studies also had similar findings that an anodal stimulation decreased phosphene thresholds (40, 41) and tACS diminished the amplitude of motor evoked potentials (42).

### 4.2 Frequency-domain results

The mainstream explanation for the mechanism of tACS was the “entrainment effect,” which indicates that endogenous oscillations during entrainment are synchronized to that of extrinsic and rhythmic stimuli (43). Prior research has provided evidence that tACS can entrain endogenous brain rhythms and increase neural synchronization in the corresponding frequency to enhance cognitive performances (20). Krause et al. found that tACS affected the firing patterns of individual neurons in conscious non-human primates (44). Later, Beliaeva and Polania supported that cortical neurons were entrained via tACS (45). However, our results were inconclusive on the entrainment effect.

The PSD analysis reached a statistical significance in neither of the montages, regardless of the power of alpha and beta bands. However, the PSD values of alpha and beta bands were all increased after tACS in every montage. Although the difference between the montages, sizes, and interaction was not statistically significant, we speculated that the effect size was underestimated by the small sample size, as were the time-domain features. Another possibility was that the changes in the surface electroencephalographic signal were too subtle to be detected. Findings of a recent animal experiment (46) on monkeys, which investigated the entrainment effect caused by low-intensity tACS, were similar to those of the current study. Electroencephalographic signals were recorded from cortical structures near the stimulation electrode to maximize the possibility for the entrainment effect to be detected at the targeted regions. From their results, approximately 10% of all recorded cells showed an entrainment effect with 1.5-mA tACS. In contrast, in the current experimental setup, only the cortical signals at the scalp surface were recorded, making the detection of the entrainment effect more difficult and indirect. Furthermore, Beliaeva and Polania found a large proportion of the recorded cells (approximately 70%) did not respond to tACS (45). Hence, at the population level (i.e., combined data from responsive and non-responsive neurons), the effects of entrainment would be apparently absent. The low prevalence of responsive cortical neurons may explain the noticeable lack of entrainment in low-intensity tACS experiments where neural activity can only be measured based on local field potentials or subcutaneous electroencephalography recordings (11).

### 4.3 Subjective sensations and blinding

Subjective sensations were common during the experiment, but most of them were well-tolerated, supporting 2-mA, 10-Hz tACS being a safe and non-invasive intervention for vision. Compared with the Oz-Cz montage, the Oz-cheek montage had a more frequent (12 vs. 15 times) and obvious (33.3% vs. 46.7% greater than mild) sensation. This outcome was expected since the cheek was directly in contact with the stimulation electrodes, while Cz was protected by the hair. In this study, visual phosphenes were also commonly reported during the active sessions. In the experiment conducted by Kanai et al., tACS was applied at Oz, with the reference electrode applied in different locations remotely from the eye to circumvent the induction of retinal phosphenes (47). Their results showed that the closer the tACS electrodes were to the retina, the more likely retinal stimulation would occur. Another interesting finding was that, when tACS was delivered in darkness, the phosphenes reported during stimulation at alpha frequencies were stronger. With the sham stimulation mimicking the first 30 s of active stimulations, the probability of guessing correctly for active stimulation (2/3) was higher than sham (1/3), indicating that the blinding in this study (especially for sham—50% accuracy) was effectively performed.

### 4.4 Montages on tACS

The superior–inferior antagonism between the two montages of the current study did not demonstrate any difference in the VEP features. Such similarity could be due to the lack of directional information in standardized VEP to differentiate the localized cortical response from tACS montages (48). Therefore, altitudinal hemifield VEP, i.e., showing only half of the checkerboard above or below the midline, may be a better method to detect the difference between Oz-Cz vs. Oz-cheek montages. It was observed that the Oz-Cz montage generated the highest VEP amplitudes, even at T0. A possible explanation was the shorter distance between Oz-Cz compared to that of Oz-cheek, with the short electrode distance inducing a stronger magnitude of neuromodulation. Another interesting finding was the different aftereffects between the two montages—the onset of tACS action of Oz-Cz was more delayed than that of Oz-cheek. As shown in Figure 2, the P100 amplitude immediately dropped at T20 for Oz-cheek, but not in Oz-Cz. The effect sustained until T40, while the tACS effect from the Oz-cheek montage started to become stronger at T40. We speculated that a quicker conductive speed of Oz-cheek benefited from less interruption from the hair.

Previous studies have illustrated how the montage type, i.e., the positions of the stimulation electrodes, altered the effect of tES on VEP amplitude; however, the evidence was limited to tDCS only (13). In particular, tDCS at Oz-Cz vs. Oz-posterior neck base montages demonstrated opposite effects on the VEP amplitude (7, 10). The difference was attributed to the amount of electricity and the location of the cortical area being stimulated by the tDCS (6, 10). Antal et al. indicated that tACS after-effects were abolished or even reversed, as observed with tDCS (49). In the current study, the tACS montages, i.e., Oz-Cz vs. Oz-cheek, had a stimulation position akin to the ventro-dorsal orientation observed in Oz-Cz vs. Oz-posterior neck base in prior research. This positioning of the stimulation may have an inhibitory effect on the VEP electrophysiological responses.

There are advantages and disadvantages for each montage. From our results, the Oz-Cz montage had fewer and weaker subjective sensations. On the other hand, Oz-cheek had a quicker onset of action and induced smaller impedances, although the efficacies of Oz-Cz and Oz-cheek were similar. It is suggested to balance among the side effects, onset of action, and impedance while choosing a suitable montage for tES interventions.

## 5 Limitations

There were several limitations in this study. First, the sample size was small, which restricted the efficacy of tACS to be detected and limited the generalization of our conclusions. Second, the experimenter was unblinded. This study adopted a single-blind design, in which the impact of the experimenter was not controlled. However, the probability of guessing correctly supported a valid blinding and a minimal effect from the experimenter. Third, there was a lack of comparison of VEP between the left and right hemispheres. A previous study indicated that the unilateral placement of the electrodes restricted the induced electric fields largely to the selected hemisphere, which could be the difference between the Oz-Cz and Oz-cheek (11). Future studies should attempt to utilize a double-blind design, replicate the findings in a larger sample size, and consider comparing differences between the left and right hemispheres.

## 6 Conclusion

The current study revealed an immediate inhibitory effect of tACS regardless of the montages. This inhibitory effect sustained in the Oz-Cz montage but faded out in other montages after 20 min. The Oz-Cz montage resulted in fewer and milder side effects, while the Oz-cheek montage produced a faster aftereffect and lower impedances. When selecting an appropriate montage, it is recommended to consider a balance between side effects, onset of action, and impedance.

## Data availability statement

The original contributions presented in the study are publicly available. This data can be found here: https://doi.org/10.17605/osf.io/khrbt.

## Ethics statement

The studies involving humans were approved by the Institutional Review Board of The Hong Kong Polytechnic University (ethical approval number: HSEARS20210713002). The studies were conducted in accordance with the local legislation and institutional requirements. The participants provided their written informed consent to participate in this study.

## Author contributions

JW: Conceptualization, Data curation, Formal analysis, Investigation, Methodology, Software, Writing – original draft, Writing – review & editing. KC: Data curation, Formal analysis, Validation, Visualization, Writing – original draft, Writing – review & editing. BT: Funding acquisition, Resources, Supervision, Writing – review & editing. HC: Methodology, Supervision, Writing – review & editing. AC: Conceptualization, Funding acquisition, Project administration, Resources, Supervision, Writing – review & editing.
