# Supplementary material for: The effect of montages of transcranial alternating current stimulation on occipital responses—a sham-controlled pilot study
Source: Front Psychiatry. 2024 Jan 24;14:1273044. doi: 10.3389/fpsyt.2023.1273044 (PMC10849049; doi:10.3389/fpsyt.2023.1273044)
Supplement: Supplementary file 1 [file Table_1.docx]

**Supplementary materials**

**Table S1.** Pattern visual evoke potential (VEP) time-domain and power spectral density features of each montage at three timepoints for two stimulus sizes

| **Time domain features** | | **Montages** (mean ± SD) | | |
| --- | --- | --- | --- | --- |
| ***N75 amplitude*** (µv) | **Size** | **Cz** | **Cheek** | **Sham** |
| T0 | **1.0 °** | -6.25 ± 2.74 | -6.33 ± 2.91 | -6.46 ± 3.09 |
| T20 |  | -6.16 ± 3.45 | -5.57 ± 2.17 | -5.77 ± 3.36 |
| T40 |  | -5.60 ± 3.42 | -5.58 ± 2.21 | -6.78 ± 3.11 |
|  |  |  |  |  |
| T0 | **0.25°** | -7.20 ± 6.19 | -6.23 ± 3.60 | -7.97 ± 5.25 |
| T20 |  | -6.90 ± 5.48 | -6.08 ± 3.59 | -7.44 ± 4.51 |
| T40 |  | -6.58 ± 5.60 | -6.62 ± 3.74 | -7.99 ± 4.06 |
| ***P100 amplitude*** (µv) |  | **Cz** | **Cheek** | **Sham** |
| T0 | **1.0 °** | 13.70 ± 6.16 | 11.97 ± 5.24 | 13.25 ± 6.12 |
| T20 |  | 12.88 ± 5.79 | 11.71 ± 5.02 | 12.33 ± 5.62 |
| T40 |  | 13.35 ± 5.89 | 12.53 ± 4.97 | 14.01 ± 5.52 |
|  |  |  |  |  |
| T0 | **0.25°** | 13.22 ± 8.31 | 8.24 ± 10.17 | 6.96 ± 13.55 |
| T20 |  | 12.32 ± 7.90 | 7.33 ± 9.56 | 6.54 ± 11.82 |
| T40 |  | 12.09 ± 8.19 | 7.41 ± 10.55 | 7.66 ± 12.19 |
| ***P100*** ***latency (ms)*** |  | **Cz** | **Cheek** | **Sham** |
| T0 | **1.0 °** | 109.4 ± 5.5 | 111.1 ± 5.5 | 109.9 ± 4.8 |
| T20 |  | 112.9 ± 7.7 | 110.8 ± 5.0 | 111.1 ± 4.7 |
| T40 |  | 112.9 ± 7.7 | 110.8 ± 5.0 | 111.1 ± 4.7 |
|  |  |  |  |  |
| T0 | **0.25°** | 116.0 ± 10.8 | 117.8 ± 6.0 | 118.0 ± 6.5 |
| T20 |  | 114.3 ± 11.0 | 118.7 ± 6.4 | 117.0 ± 6.1 |
| T40 |  | 115.3 ± 11.0 | 117.7 ± 6.4 | 116.7 ± 6.6 |
| **Spectral domain features** | | | | |
| ***log Alpha band*** |  | **Cz** | **Cheek** | **Sham** |
| T0 | **1.0 °** | 6.88 ± 0.48 | 6.79 ± 0.43 | 6.87 ± 0.45 |
| T20 |  | 11.62 ± 1.27 | 11.41 ±0.74 | 11.32 ± 0.94 |
| T40 |  | 11.37 ± 1.04 | 11.59 ± 0.77 | 11.23 ± 0.69 |
|  |  |  |  |  |
| T0 | **0.25°** | 6.72 ±0.60 | 6.67 ±0.44 | 6.73 ± 0.49 |
| T20 |  | 10.94 ± 0.75 | 11.05 ±1.14 | 10.51 ± 0.83 |
| T40 |  | 11.07 ± 0.76 | 11.24 ± 1.16 | 10.64 ± 1.32 |
| ***log Beta band*** |  | **Cz** | **Cheek** | **Sham** |
| T0 | **1.0 °** | 6.77 ± 0.56 | 6.67 ± 0.51 | 6.75 ± 0.50 |
| T20 |  | 11.47 ± 1.18 | 11.26 ± 0.80 | 11.15 ± 0.98 |
| T40 |  | 11.37 ± 0.96 | 11.42 ± 0.82 | 11.09 ± 0.76 |
|  |  |  |  |  |
| T0 | **0.25°** | 6.69 ± 0.52 | 6.66 ± 0.43 | 6.68 ± 0.50 |
| T20 |  | 10.91 ± 0.72 | 11.02 ± 1.03 | 10.45 ± 0.78 |
| T40 |  | 11.03 ± 0.71 | 11.22 ± 1.17 | 10.60 ± 1.23 |
