# Supplementary figures and images for: The effect of montages of transcranial alternating current stimulation on occipital responses—a sham-controlled pilot study
Source: Front Psychiatry. 2024 Jan 24;14:1273044. doi: 10.3389/fpsyt.2023.1273044 (PMC10849049; doi:10.3389/fpsyt.2023.1273044)

**Supplementary materials**


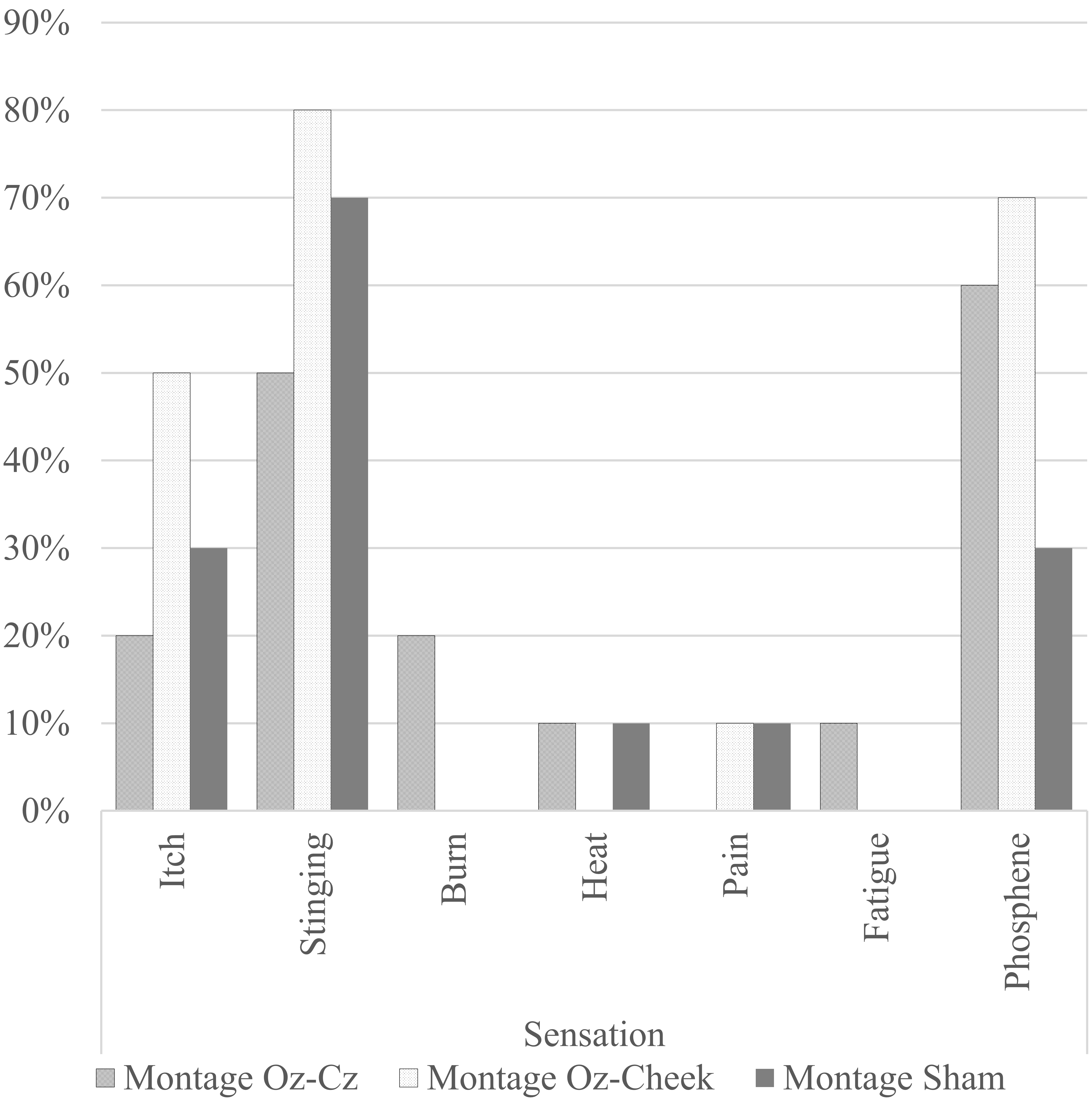


**Figure S1.** Proportion of subjective sensation reports.

Supplement: Supplementary file 2 [file Data_Sheet_1.docx]
